# Transcriptional Responses of Pseudomonas aeruginosa to Inhibition of Lipoprotein Transport by a Small Molecule Inhibitor

**DOI:** 10.1128/JB.00452-20

**Published:** 2020-11-19

**Authors:** Christian Lorenz, Thomas J. Dougherty, Stephen Lory

**Affiliations:** a Department of Microbiology, Blavatnik Institute, Harvard Medical School, Boston, Massachusetts, USA; Geisel School of Medicine at Dartmouth

**Keywords:** LolCDE, *Pseudomonas*, lipoprotein transport, molecular inhibitor, transcriptome

## Abstract

Lipoprotein transport from the inner to the outer membrane, carried out by the Lol machinery, is essential for the biogenesis of the Gram-negative cell envelope and, consequently, for bacterial viability. Recently, small molecule inhibitors of the Lol system in Escherichia coli have been identified and shown to inhibit the growth of this organism by interfering with the function of the LolCDE complex. Analysis of the transcriptome of E. coli treated with one such molecule (compound 2) revealed that a number of envelope stress response pathways were induced in response to LolCDE inhibition. However, Pseudomonas aeruginosa is refractory to inhibition by the same small molecule, but we could demonstrate that E. coli *lolCDE* could be substituted for the P. aeruginosa orthologues, where it functions in the correct transport of *Pseudomonas* lipoproteins, and the cells are inhibited by the more potent compound 2A. In the present study, we took advantage of the functionality of E. coli LolCDE in P. aeruginosa and determined the P. aeruginosa transcriptional response to LolCDE inhibition by compound 2A. We identified key genes that responded to LolCDE inhibition and also demonstrated that the same genes appeared to be affected by genetic depletion of the native P. aeruginosa LolCDE proteins. Several of the major changes were in an upregulated cluster of genes that encode determinants of alginate biosynthesis and transport, and the levels of alginate were found to be increased either by treatment with the small molecule inhibitor or upon depletion of native LolCDE. Finally, we tested several antibiotics with differing mechanisms of action to identify potential specific reporter genes for the further development of compounds that would inhibit the native P. aeruginosa Lol system.

**IMPORTANCE** A key set of lipoprotein transport components, LolCDE, were inhibited by both a small molecule as well as genetic downregulation of their expression. The data show a unique signature in the Pseudomonas aeruginosa transcriptome in response to perturbation of outer membrane biogenesis. In addition, we demonstrate a transcriptional response in key genes with marked specificity compared to several antibiotic classes with different mechanisms of action. As a result of this work, we identified genes that could be of potential use as biomarkers in a cell-based screen for novel antibiotic inhibitors of lipoprotein transport in P. aeruginosa.

## Abstract (precis)

A key set of lipoprotein transport components, LolCDE, were inhibited by both a small molecule as well as genetic downregulation of their expression. The data show a unique signature in the Pseudomonas aeruginosa transcriptome in response to perturbation of outer membrane biogenesis. In addition, we demonstrate a transcriptional response in key genes with marked specificity compared to several antibiotic classes with different mechanisms of action. As a result of this work, we identified genes that could be of potential use as biomarkers in a cell-based screen for novel antibiotic inhibitors of lipoprotein transport in P. aeruginosa.

## INTRODUCTION

The outer membrane (OM) of Gram-negative bacteria contains a number of lipoproteins that are essential for cell envelope integrity and are key components of numerous nanomachines, including the peptidoglycan biosynthesis apparatus, the flagellar basal body, and various transport systems for proteins, lipopolysaccharide, and antibiotics (1–5). Disruption of the lipoprotein transport system inhibits the assembly of these critical molecules into the outer membrane, compromising cell envelope integrity and function, leading to the loss of cell viability (6–11).

In Gram-negative bacteria, a substantial fraction of the lipoproteins are bound to the OM. A dedicated lipoprotein localization machinery directs their targeting to the OM, which includes extraction from the inner cytoplasmic membrane (IM), transport across the periplasm, and incorporation into the OM in a functional form (2, 12). In gammaproteobacteria, the lipoprotein transport pathway consists of a LolCDE ATP-binding cassette transporter responsible for the recognition and release of the OM-targeted lipoproteins from the IM and directing them into a complex with the periplasmic molecular chaperone LolA. In turn, LolA transports the nascent lipoprotein across the periplasm (3, 4, 13). The final step in lipoprotein biogenesis is their transfer from LolA into the OM; this process is facilitated by the OM lipoprotein LolB (14, 15). Recent evidence has emerged suggesting that, at least in Escherichia coli, an alternative pathway utilizes LolCDE but not LolA and LolB (16).

Previously, we described the transcriptional responses of Escherichia coli to inhibition of lipoprotein transport to the OM (17) by a novel, small molecule inhibitor referred to as compound 2 (18). This inhibitor interacts with E. coli LolCDE, and amino acid substitution mutants displaying high-level resistance are located in LolC or LolE. In transcriptome sequencing (RNA-seq) transcription studies in E. coli, the primary responses to the inhibition of LolCDE were in the CpxA/R, σ^S^, and regulator of capsular synthesis (RCS) envelope stress response systems (17, 19–21). Neither the RpoE (σ^E^)-, RpoH (σ^32^)-, nor BaeSR-controlled genes were upregulated by compound 2 inhibition (22).

In a study of lipoprotein transport in Pseudomonas aeruginosa, we found that the LolCDE system from E. coli could replace the native *Pseudomonas* LolCDE (23). Placing the E. coli *lolCDE* genes in the P. aeruginosa chromosome at the *ctx* phage site and subsequently deleting the native *lolCDE* genes resulted in cells that showed growth kinetics comparable to those of the wild type, had normal cell morphology, and were found to localize several tested proteins to the correct membrane compartments (23). In addition, the E. coli *lolCDE* genes inserted into the P. aeruginosa *ctx* site were under the control of the arabinose promoter, and this strain (P. aeruginosa PAO1 Δ*mexAB-oprM* Δ*lolCDE_PAO1_ ctx*::*lolCDE_E.coli_*) was arabinose dependent for growth. The successful substitution of the E. coli *lolCDE* genes for the P. aeruginosa orthologues was somewhat unexpected as previous studies suggested that these two bacteria employ different sorting signals for lipoproteins destined for the IM or OM, and these were recognized by the LolCDE complex (24, 25).

In the case of wild-type P. aeruginosa, neither compound 2 nor a more potent derivative, compound 2A (26) (see Fig. S1 in the supplemental material), was lethal. This observation was not unexpected as there are important differences between E. coli and P. aeruginosa in their respective LolCDE amino acid sequences, and key differences are located at the sites of some of the LolCDE compound 2-resistant E. coli mutants. When the above-described LolCDE replacement strain was used, the P. aeruginosa strain became susceptible to the inhibitor due to the reliance on the compound-sensitive E. coli *lolCDE* genes for viability.

The observations that the E. coli LolCDE system could support P. aeruginosa growth and normal lipoprotein transport, along with the ability of compound 2A to inhibit the replacement E. coli system, suggested that it would be possible to define the P. aeruginosa transcriptional responses to inhibition of Lol-mediated lipoprotein transport by compound 2A, employing the strain expressing the susceptible E. coli LolCDE. In this report, we describe the effect of compound 2A on the P. aeruginosa transcriptome, where we observe a transcriptional response that is different from that in E. coli treated with the same compound. The expression of a set of genes in this P. aeruginosa strain expressing heterologous *lolCDE* was the same as that in a strain where the levels of its native LolCDE were reduced by limiting the expression of the corresponding genes. In addition, we found several genes that appear to specifically respond to compound 2A inhibition of LolCDE while remaining unperturbed by several antibiotics with differing mechanisms of action.

## RESULTS

### Transcriptional responses of Pseudomonas aeruginosa to LolCDE inhibition by a small molecule inhibitor.

**FIG 1:**
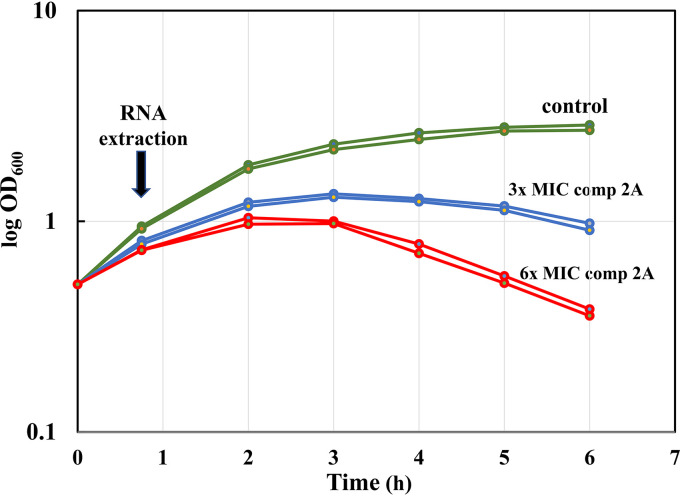
Growth of the P. aeruginosa PAO1 Δ*mexAB-oprM* Δ*lolCDE_PAO1_ ctx*::*lolCDE_E.coli_* strain. The cells were either untreated (control) (green) or treated with 3× MIC (48 μg/ml) (blue) or 6× MIC (96 μg/ml) (red) of compound 2A. Duplicate individual cultures under each condition were employed. Time zero represents the cell density at the initial time of compound 2A exposure. The time point indicated at 45 min of compound 2A exposure is where the RNA samples were taken for RNA-seq. Extended incubation times led to cell death and lysis.

P. aeruginosa is not naturally susceptible to either compound 2 or 2A due to overall modest amino acid sequence identity with LolC and LolE from E. coli (LolC, 38.9% identity; LolE, 35.6% identity), including key residues found at the sites of compound 2-resistant E. coli mutants (17, 18). However, the strain described previously where the wild-type E. coli *lolCDE* genes can function in P. aeruginosa lipoprotein transport becomes susceptible to compounds 2 and 2A (23). Compound 2A is similar to compound 2, and it is more potent; however, it is a substrate for the MexAB-OprM efflux pump. Consequently, a Δ*mexAB-oprM* strain was employed in this study. The MIC of this P. aeruginosa strain with the E. coli *lolCDE* genes for compound 2A is 16 μg/ml. Figure 1 shows the growth curves of the P. aeruginosa PAO1 Δ*mexAB-oprM* Δ*lolCDE_PAO1_ ctx*::*lolCDE_E.coli_* strain at 3× (48 μg/ml) and 6× (96 μg/ml) the MIC of compound 2A along with the untreated control. Two duplicate cultures were used, and the time of sampling for RNA isolation after compound addition was at 45 min, approximately 1 doubling at an optical density (OD) of 0.5, as indicated in Fig. 1. Following extended incubation, the cultures exposed to the compound underwent cell death and lysis at both 3× and 6× MIC.

Following RNA extraction with hot acid phenol, DNase treatment, and rRNA depletion, the RNA was converted to cDNA and sequenced on an Illumina NextSeq 500 platform. The number of reads for the individual samples ranged from 23,387,502 to 27,335,904 (see Table S1 in the supplemental material). Tables 1 and 2 show genes significantly upregulated or downregulated compared to the control at the two concentrations of compound 2A exposure. Table 1 lists the transcripts that were upregulated by 10-fold or more with at least one of the two compound exposure concentrations. Most prominently upregulated are numerous genes associated with alginate biosynthesis, osmotic regulation, and lipotoxin F and a number of transcripts annotated as encoding hypothetical proteins. There were fewer transcripts downregulated by 3-fold or more in Table 2, and the genes encoding the components of flagella and type IV pili are well represented in this class. The complete sets of RNA-seq transcriptional data are available in Tables S2 and S3 in the supplemental material.

**TABLE 1:** RNA levels of genes significantly upregulated (10-fold) by compound 2A

Locus tag	Gene	Fold change vs the control^*a*^		Functional assignment
		3× MIC	6× MIC	
PA3540	*algD*	150.5	107.4	GDP-mannose 6-dehydrogenase AlgD
PA3546	*algX*	48.3	35.4	Alginate biosynthesis protein AlgX
PA3547	*algL*	39.3	31.3	Poly(β-d-mannuronate) lyase precursor AlgL alginate lyase (AlgL)
PA2146		38.4	102.0	Conserved hypothetical protein
PA3551	*algA*	38.1	33.0	Phosphomannose isomerase alginate biosynthesis protein AlgA
PA3541	*alg8*	25.8	18.5	Alginate biosynthesis protein Alg8
PA3550	*algF*	24.8	22.2	Alginate *O*-acetyltransferase AlgF
PA3544	*algE*	23.4	19.5	Alginate production outer membrane protein AlgE precursor
PA2168		21.4	29.0	Hypothetical protein
PA3549	*algJ*	19.4	15.9	Alginate *O*-acetyltransferase AlgJ
PA3691		18.2	31.5	Hypothetical protein
PA3542	*alg44*	17.7	14.2	Alginate biosynthesis protein Alg44
PA1471		17.7	22.0	Hypothetical protein
PA3692	*lptF*	16.7	31.1	Lipotoxin F, LptF
PA1323		15.7	26.4	Hypothetical protein
PA1324		15.2	23.8	Hypothetical protein
PA2167		15.1	21.7	Hypothetical protein
PA0737		14.3	14.7	Hypothetical protein
PA2169		14.2	23.7	Hypothetical protein
PA2562		13.2	17.8	Hypothetical protein
PA0059	*osmC*	13.2	18.1	Osmotically inducible protein OsmC
PA2414		12.7	15.7	l-Sorbosone dehydrogenase
PA2176		12.6	13.9	Hypothetical protein
PA3548	*algI*	12.5	9.6	Alginate *O*-acetyltransferase AlgI
PA2171		12.2	17.9	Hypothetical protein
PA4876	*osmE*	12.2	18.7	Osmotically inducible lipoprotein OsmE
PA1283		12.1	11.7	Probable transcriptional regulator
PA2173		11.9	18.0	Hypothetical protein
PA2170		11.5	15.8	Hypothetical protein
PA5212		11.4	17.2	Hypothetical protein
PA1592		11.2	10.0	Hypothetical protein
PA0355	*pfpI*	11.1	17.6	Protease PfpI
PA2754		10.5	14.9	Conserved hypothetical protein
PA1281	*cobV*	10.5	11.1	Cobalamin (5′-phosphate) synthase
PA4154		10.5	9.2	Conserved hypothetical protein
PA3543	*algK*	9.9	7.2	Alginate biosynthetic protein AlgK precursor
PA2815		9.8	12.8	Probable acyl coenzyme A dehydrogenase
PA2172		9.7	13.2	Hypothetical protein
PA2415		9.5	11.3	Hypothetical protein
PA2141		9.3	11.3	Hypothetical protein
PA3404		9.3	11.3	Probable outer membrane protein precursor
PA2717	*cpo*	9.0	12.0	Chloroperoxidase precursor
PA4345		8.9	11.0	Hypothetical protein
PA2159		8.9	18.7	Conserved hypothetical protein
PA2163		8.6	13.7	Hypothetical protein
PA2161		8.4	15.1	Hypothetical protein
PA4877		8.3	10.5	Hypothetical protein
PA2180		8.3	14.0	Hypothetical protein
PA2134		8.3	13.5	Hypothetical protein
PA5424		8.2	12.1	Conserved hypothetical protein
PA3231		8.2	12.8	Hypothetical protein
PA3040		8.1	11.3	Conserved hypothetical protein
PA2149		8.0	11.5	Hypothetical protein
PA4880		7.5	14.2	Probable bacterioferritin
PA2148		7.5	10.4	Conserved hypothetical protein
PA2143		7.4	12.3	Hypothetical protein
PA0567		7.2	11.1	Conserved hypothetical protein

a Values represent the fold upregulation of expression at the two compound 2A concentrations at 3× and 6× MIC relative to untreated control cells.

**TABLE 2:** RNA levels of genes downregulated 3-fold or more by compound 2A

Locus tag	Gene	Fold change vs the control^*a*^		Functional assignment
		3× MIC	6× MIC	
PA5139		−7.0	−13.8	Hypothetical protein
PA5138		−6.4	−8.2	Hypothetical protein
PA1913		−5.9	−2.9	Hypothetical protein
PA0277		−5.8	−9.8	Conserved hypothetical protein
PA5042	*pilO*	−5.3	−5.5	Type 4 fimbrial biogenesis protein PilO
PA5041	*pilP*	−5.3	−5.1	Type 4 fimbrial biogenesis protein PilP
PA5043	*pilN*	−5.2	−5.6	Type 4 fimbrial biogenesis protein PilN
PA5040	*pilQ*	−4.9	−5	Type 4 fimbrial biogenesis outer membrane protein PilQ precursor
PA1867	*xphA*	−4.8	−11.6	XphA
PA0952		−4.4	−5.9	Hypothetical protein
PA1081	*flgF*	−4.3	−5.6	Flagellar basal body rod protein FlgF
PA5137		−4.3	−4.7	Hypothetical protein
PA1657	*hsiB2*	−4.3	−3.7	HsiB2
PA3912		−4.2	−3.1	Conserved hypothetical protein
PA0563		−4.2	−4.9	Conserved hypothetical protein
PA1868	*xqhA*	−4.2	−6.6	Secretion protein XqhA
PA1659	*hsiF2*	−4.2	−3.2	HsiF2
PA1082	*flgG*	−4.1	−3.9	Flagellar basal body rod protein FlgG
PA1077	*flgB*	−4.0	−4	Flagellar basal body rod protein FlgB
PA2760	*oprQ*	−4.0	−4.6	OprQ
PA1452	*flhA*	−4.0	−4.8	Flagellar biosynthesis protein FlhA
PA1658	*hsiC2*	−3.9	−3.3	HsiC2
PA2463		−3.7	−3.4	Hypothetical protein
PA5044	*pilM*	−3.7	−4.1	Type 4 fimbrial biogenesis protein PilM
PA0126		−3.7	−5	Hypothetical protein
PA5033		−3.7	−5	Hypothetical protein
PA1100	*fliE*	−3.7	−4.3	Flagellar hook-basal body complex protein FliE
PA4525	*pilA*	−3.6	−4.5	Type 4 fimbrial precursor PilA type IV pilin
PA2783	*mep72*	−3.6	−4.6	Mep72
PA3278		−3.6	−3.2	Hypothetical protein
PA0086	*tagJ1*	−3.6	−4.8	TagJ1
PA1098	*fleS*	−3.6	−4.4	Two-component sensor
PA4726.1	*P36*	−3.5	−3.5	P36
PA1441		−3.4	−4	Putative flagellar hook-length control protein FliK
PA1099	*fleR*	−3.4	−4	Two-component response regulator
PA1556	*ccoO2*	−3.4	−3.8	Cytochrome *c* oxidase, *cbb*_3_ type, CcoO subunit
PA0047		−3.4	−4.3	Hypothetical protein
PA2782	*bamI*	−3.4	−3.9	Biofilm-associated metzincin inhibitor, BamI hypothetical protein
PA0087	*tssE1*	−3.4	−4.8	TssE1
PA1101	*fliF*	−3.4	−3.9	Flagellar M-ring outer membrane protein precursor
PA1083	*flgH*	−3.4	−2.8	Flagellar L-ring protein precursor FlgH
PA0958	*oprD*	−3.4	−4.1	Basic peptide and imipenem outer membrane porin OprD
PA0088	*tssF1*	−3.4	−4.4	TssF1
PA2784		−3.3	−2.8	Hypothetical protein
PA1663	*sfa2*	−3.3	−3.2	Sfa2
PA1078	*flgC*	−3.3	−4.1	Flagellar basal body rod protein FlgC
PA1555	*ccoP2*	−3.3	−3.8	Cytochrome *c* oxidase, *cbb*_3_ type, CcoP subunit
PA1661	*hsiH2*	−3.3	−3.2	HsiH2
PA0089	*tssG1*	−3.3	−4.4	TssG1
PA0046		−3.3	−4.4	Hypothetical protein
PA1084	*flgI*	−3.2	−3.5	Flagellar P-ring protein precursor FlgI
PA2539		−3.2	−4.1	Conserved hypothetical protein
PA0085	*hcp1*	−3.2	−3.9	Hcp1
PA3911		−3.2	−3.5	Conserved hypothetical protein
PA1079	*flgD*	−3.2	−3.5	Flagellar basal body rod modification protein FlgD flagellar hook cap
PA4524.1		−3.2	−4.3	tRNA-Thr
PA5472		−3.1	−4.3	Hypothetical protein
PA1662	*clpV2*	−3.1	−3	ClpV2
PA2450		−3.1	−3.8	Hypothetical protein
PA0411	*pilJ*	−3.1	−3.5	Twitching motility protein PilJ type 4 fimbrial biogenesis protein PilJ
PA1967		−3.0	−3.9	Hypothetical protein

a Values represent the fold downregulation of expression relative to the untreated control.

Examining the data (Table 3) for the known two-component regulator systems of virulence and antibiotic responses in P. aeruginosa, it was possible to identify changes in the regulation of systems associated with alginate (upregulated) and type IV pili and flagella (both downregulated). These are very consistent with the other transcript expression changes in genes associated with these regulators upon compound 2A addition.

**TABLE 3:** Effect of compound 2A (3× and 6× MIC) on known regulatory systems^*a*^

Locus tag	Gene	Fold change with compound 2A		Function(s)^*b*^
		3× MIC	6× MIC	
PA0034		−1.4	−1.5	R
**PA0408**	***pilG***	−2.5	−2.7	
**PA0409**	***pilH***	−2.5	−2.7	
PA0463	*creB*	1.6	1.6	R
PA0464	*creC*	1	−1.1	S
PA0929	*pirR*	1.8	1.8	R
PA0930	*pirS*	1.5	1.7	S
**PA1099**	***fleR***	−3.6	−4.4	R
**PA1098**	***fleS***	−3.4	−4	S
PA1157		−1.7	−2.2	R
PA1158		−1.6	−1.8	S
PA1179	*phoP*	1.1	−1.2	R
PA1180	*phoQ*	1	−1.3	S
PA1135		1.1	2.3	
PA1136		−1.1	1.6	
PA2523	*czcR*	1.4	2.9	R
PA2524	*czcS*	1.1	1.4	S
PA2586	*gacA*	−1.3	−1.3	R
PA2686	*pfeR*	−1.1	1	R
PA2687	*pfeS*	−1.2	−1.2	S
PA2809	*copR*	1	−1.1	R
PA2810	*copS*	−1.1	−1.1	S
PA3045	*rocA2*	−1.3	−1.4	R
PA3191	*gtrS*	−1.1	−1.2	S
PA3192	*gltR*	1.2	1	R
PA3204		1.8	1.8	R
PA3206		1	2.8	S
PA3346		1.4	1.4	R
PA3702	*wspR*	−1.1	1	R
PA3704	*wspE*	−1.3	1	S
PA3878	*narX*	−2.3	−1.9	S
PA3879	*narL*	−1.6	−1.2	R
PA3947	*rocR*	1.5	1.4	R
PA3948	*rocA1*	−1.4	−1.6	
PA4293	*pprA*	−1.1	−1.1	S
PA4296	*pprB*	−1.3	1.3	R
PA4396		−1.2	−1.4	R
PA4546	*pilS*	−1.6	−1.6	S
PA4547	*pilR*	−1.4	−1.3	R
PA4725	*cbrA*	−1.2	−1.1	S
PA4726	*cbrB*	1	1	R
PA4776	*pmrA*	1.9	2.6	R
PA4777	*pmrB*	1.7	2.2	S
PA4959	*fimX*	−1.7	−1.6	R
PA5261	*algR*	4.9	4.9	R
PA5262	*fimS*	2.8	2.7	S
PA5360	*phoB*	1	−1.2	R
PA3561	*phoR*	−1.1	−1.3	S
PA5483	*algB*	5.2	5.2	R
PA5484	*kinB*	4.2	4.4	S
PA0928	*gacS*	−1.3	−1.2	S/R
PA1611		−1.4	1.1	S
PA3044	*rocS2*	−1.4	1	S
PA3946	*rocS1*	−1.2	−1.1	S
PA3974	*ladS*	−2.5	−2.5	S
PA4112		1	1.1	S/R
PA4856	*retS*	−1.6	−1.9	S
PA4982		−1.1	−1.1	S

a The two-component regulator genes indicated in boldface type are associated with the downregulation of pilus and flagellum expression. Two-component regulator genes indicated by underlining are associated with the increased expression of alginate and related pathways. Changes are relative to untreated control cells.

b S, sensor kinase; R, response regulator.

### Alginate production in response to compound 2A.

**FIG 2:**
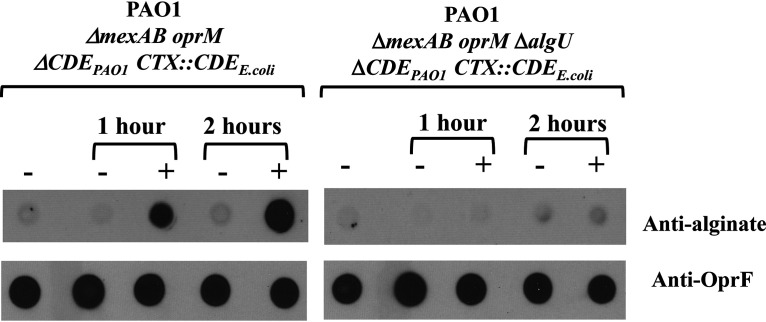
Dot blots of cell lysates with antialginate antibody and anti-OprF antibody as a control. Treatment of cells with compound 2A for the indicated times is signified by +. On the left, it is clear that compound 2A caused a significant increase in alginate expression at both 1 and 2 h. Deletion of the *algU* gene abrogated expression. The OprF antiserum blots indicated similar levels of cell extraction in all cases.

A notable finding in the RNA-seq analysis was an increase, in response to compound 2A, in the level of transcripts from the cluster of genes responsible for the biosynthesis, modification, and export of alginate. Because treatment with compound 2A is lethal, the alginate product could not be detected by the growth of mucoid colonies. Antibodies to alginate were available, offering the possibility of examining alginate production (Fig. 2). P. aeruginosa PAO1 Δ*mexAB-oprM* Δ*lolCDE_Pa_ ctx*::*lolCDE_E.coli_* cells were treated for 1 or 2 h with compound 2A, and normalized lysates were prepared and analyzed by dot blot immunoassays with antialginate as well as antibodies against the outer membrane protein OprF. The results clearly show that alginate production was increased in response to treatment of the bacteria with compound 2A. A matching control using a strain with the *algU* gene deleted did not exhibit increased levels of alginate (Fig. 2). These results indicate that inhibition of lipoprotein transport by compound 2A affects alginate production via a regulatory mechanism acting through the AlgU-mediated control of the alginate biosynthetic operon.

### Specificity of the response to compound 2A.

**FIG 3:**
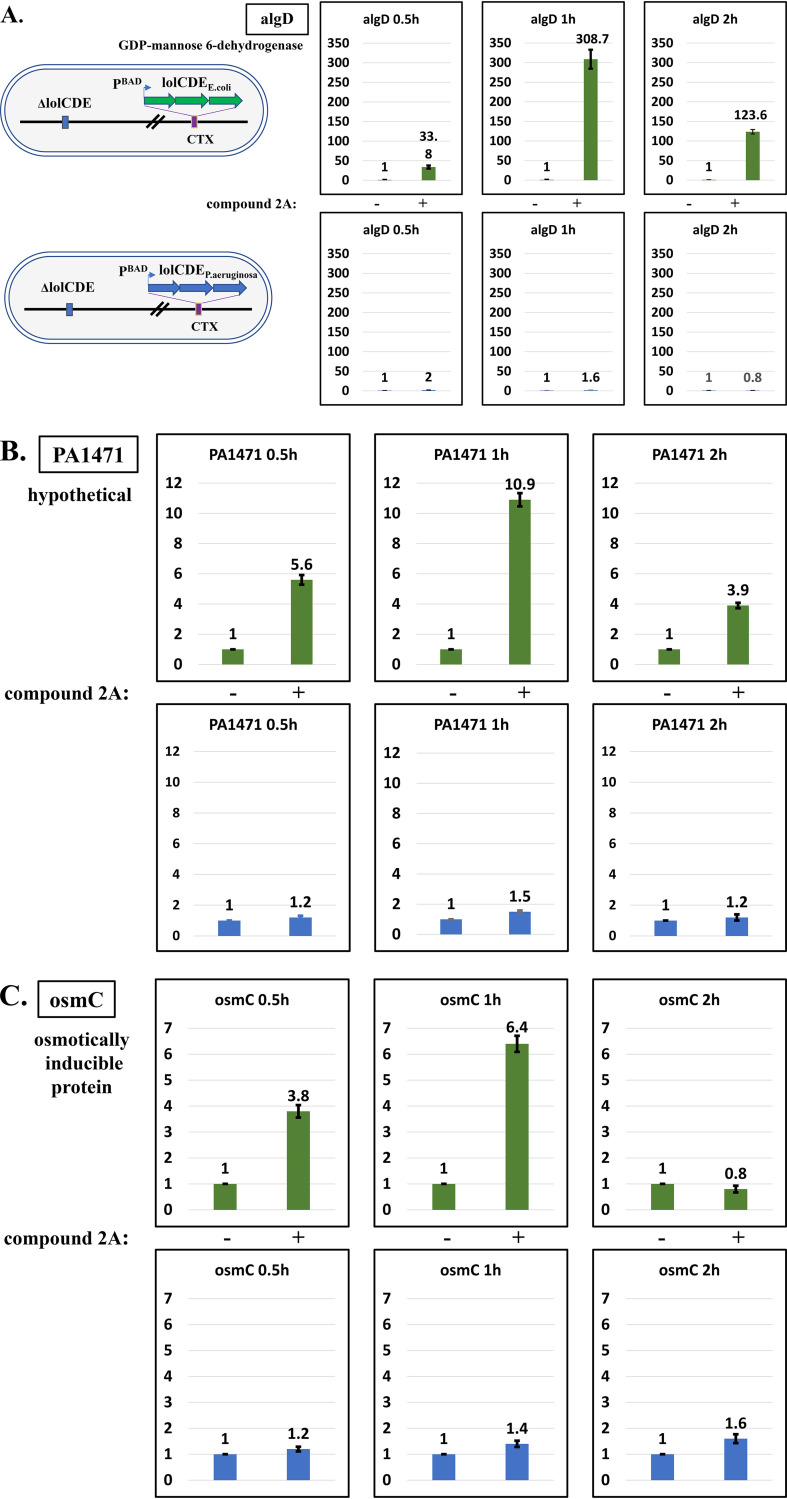

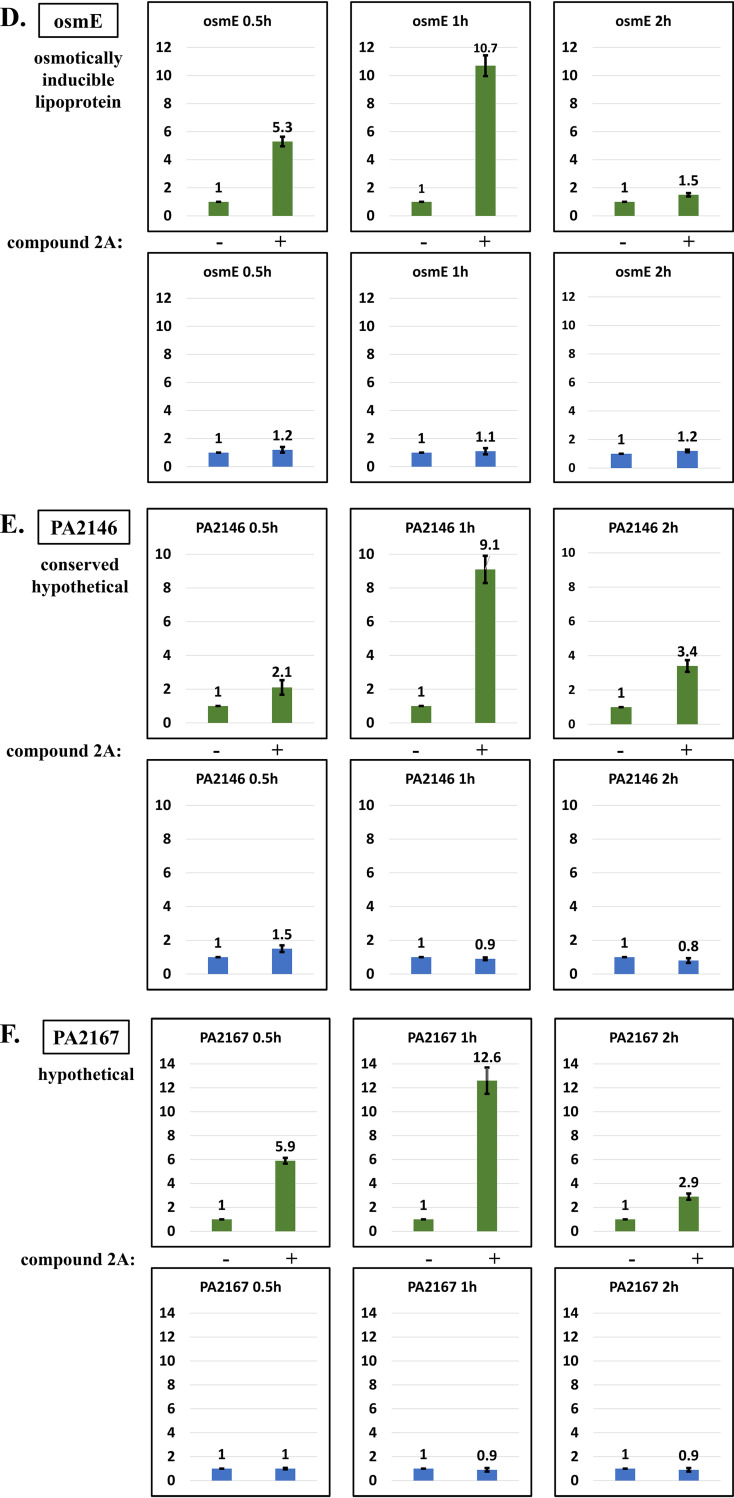

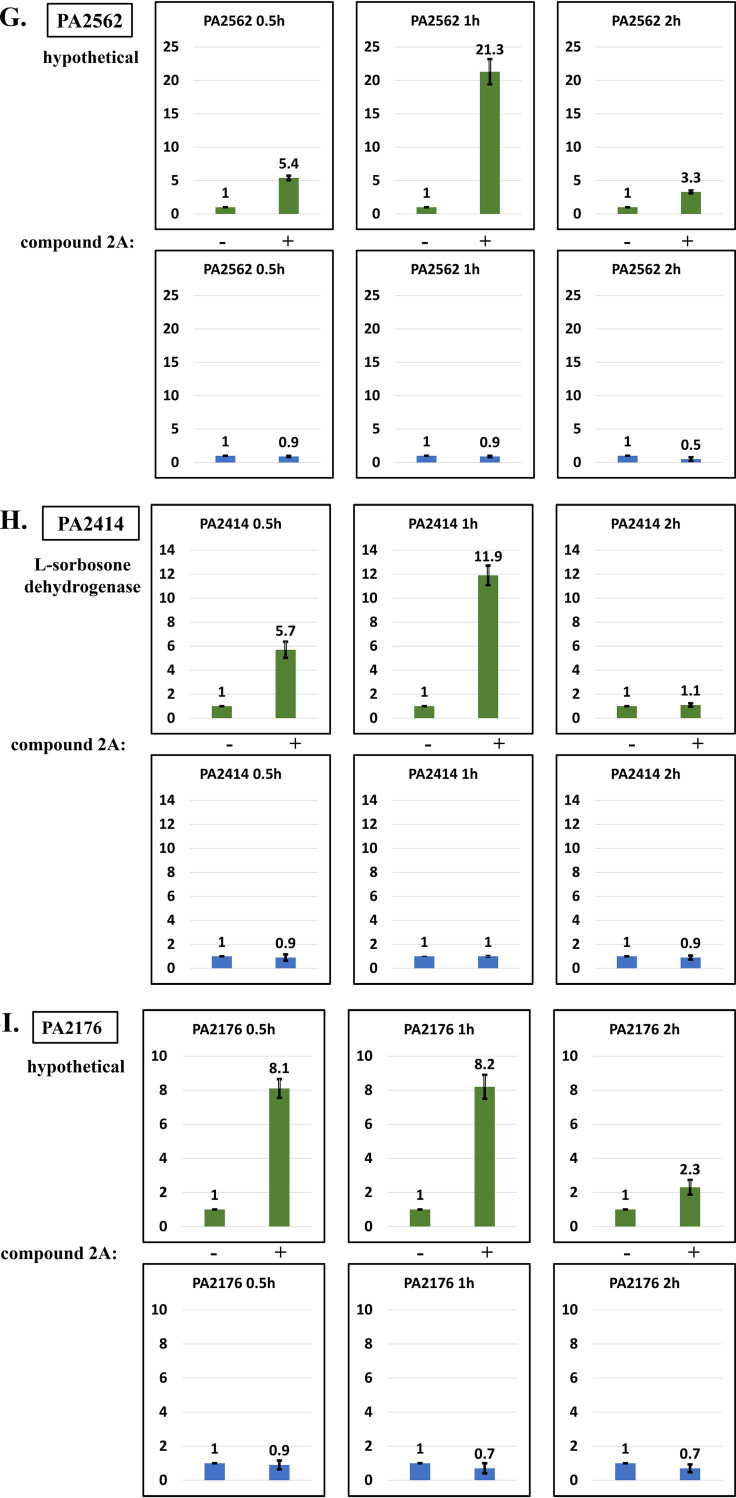

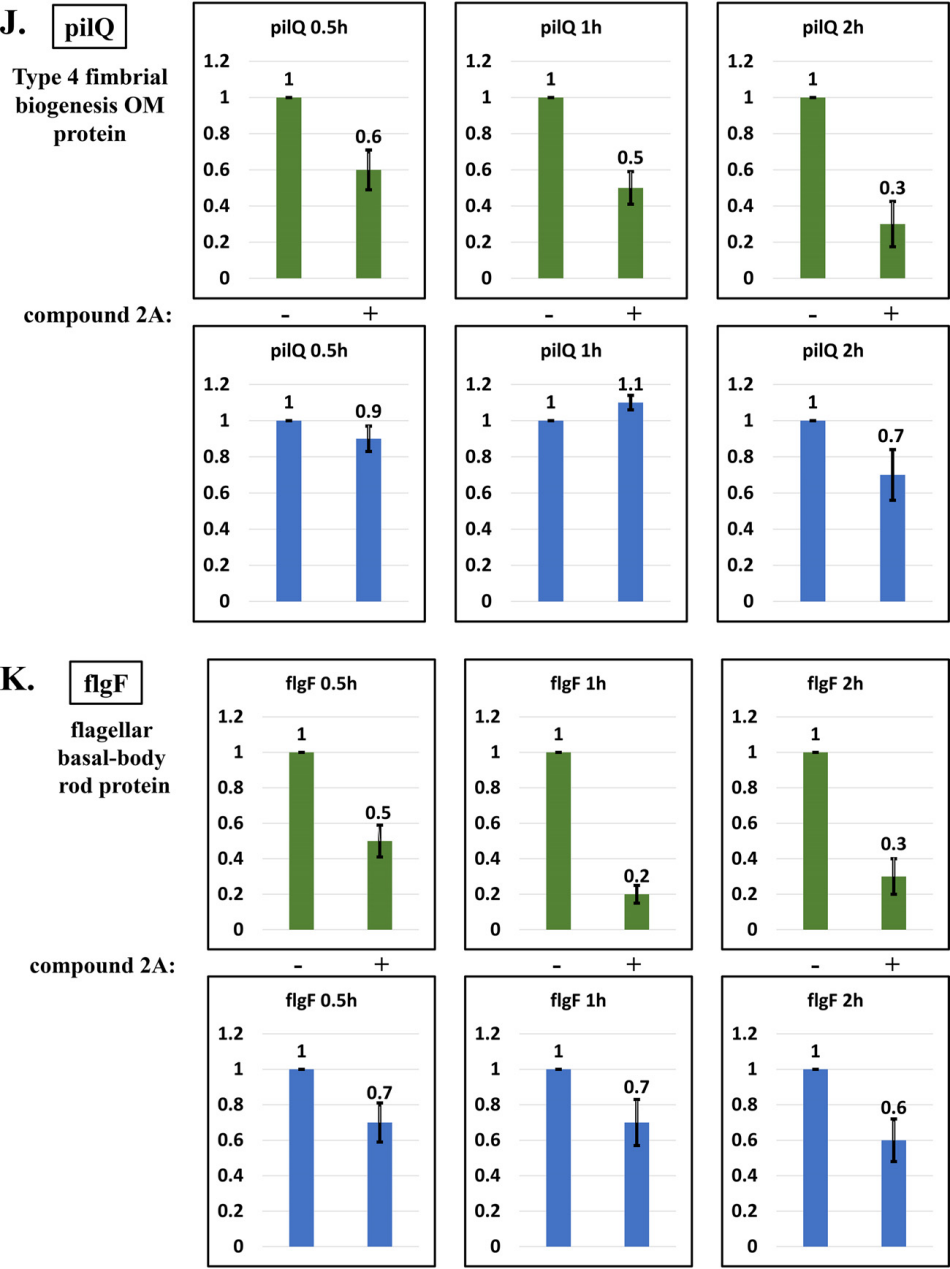
Dependence of the regulation of selected genes by compound 2A on the presence of the E. coli *lolCDE* genes. Panel A illustrates the two P. aeruginosa strains with E. coli *lolCDE* genes in the *ctx* site (green bars) or P. aeruginosa *lolCDE* genes (blue bars) in the *ctx* site. Levels of gene expression were measured by RT-qPCR at 3 time intervals, 0.5, 1, and 2 h. Untreated control cells and cells treated with 16 μg/ml (1× MIC) are indicated by – and + for both strains. The values are expressed as fold changes over the values for the untreated controls, and the standard deviations from independent duplicate experiments on different days are indicated by vertical bars. Panels A through I show genes that were upregulated more than 5-fold, and panels J and K below the line, show genes identified as being downregulated. Changes tended to peak at around 1 h and subsequently declined, most likely due to decreased viability from compound 2A. In all cases, the no effects of compound 2A were observed with native P. aeruginosa *lolCDE*-containing cells.

As mentioned above, the native P. aeruginosa LolCDE is refractory to the effects of compound 2A due to key amino acid differences, and it is only by substituting E. coli LolCDE that P. aeruginosa becomes susceptible to this inhibitor of lipoprotein transport. Therefore, by comparing P. aeruginosa responses to compound 2A with the two LolCDE versions, it can be established that the responses observed were through susceptible LolCDE inhibition. Again, the transcripts of genes exhibiting the most responses to compound 2A were tested using reverse transcription-quantitative PCR (RT-qPCR) after exposure to 1× MIC of compound 2A. Figure 3 illustrates the results for 3 different time intervals. It is clear that only in the case of P. aeruginosa cells with the susceptible E. coli version of LolCDE did we observe changes in transcript levels. This indicates that the transcriptional changes are a direct consequence of the effect of compound 2A on the susceptible E. coli LolCDE. Change magnitudes mostly peaked at 1 h of compound treatment, diminishing with prolonged exposure time due to subsequent growth impairment and viability loss.

**FIG 4:**
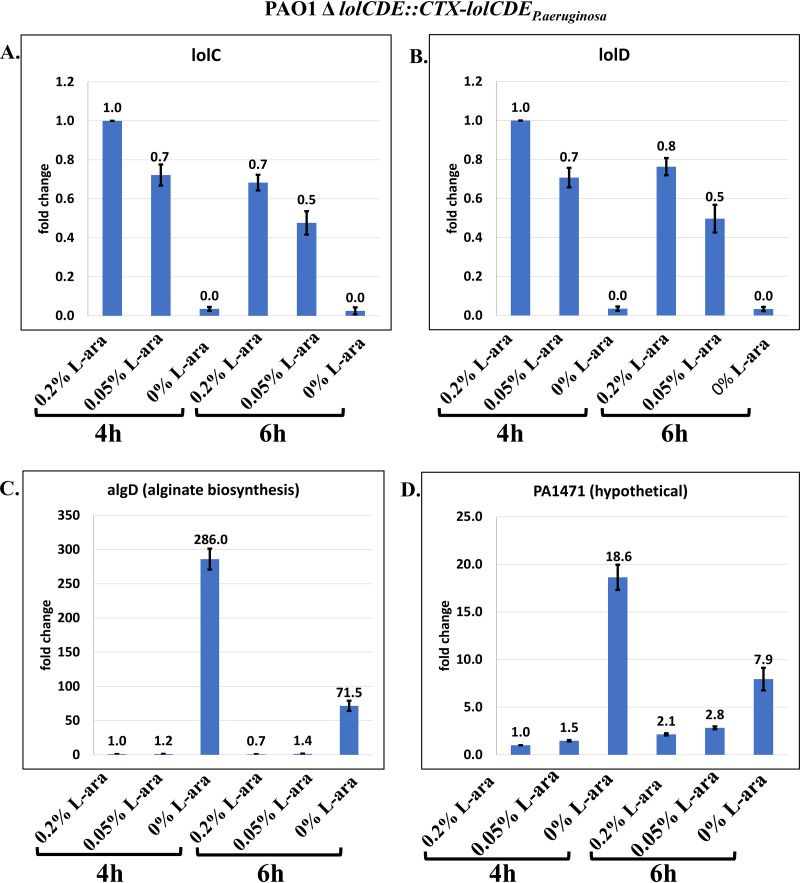
Expression level changes due to depletion of LolCDE in P. aeruginosa. The *Pseudomonas* LolCDE genes under the control of the arabinose promoter system were placed in the *ctx* phage attachment site. Subsequently, the native LolCDE was deleted from the P. aeruginosa chromosome, thus making the cells arabinose dependent for viability. After growing the cells in the presence of sufficient arabinose (0.2%), downregulation (0.05%) or depletion (no arabinose) was used, and the levels of several genes over time were measured by RT-qPCR. The numbers are averages from two independent experiments, with standard deviations indicated by vertical bars. The levels of *lolC* and *lolD* transcripts were determined over time, as were those for *algD* and PA1471. The 6-h depletion levels are lower, reflecting the increased loss of viability at that time point. Additional 4-h values are shown in Fig. S2 in the supplemental material.

**FIG 5:**
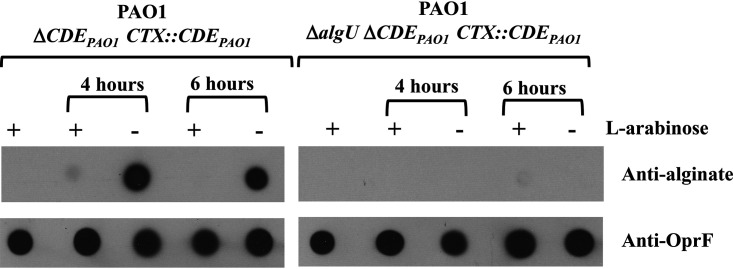
Production of alginate by depletion of native P. aeruginosa LolCDE detected by antibody dot blotting. The P. aeruginosa strain in which the chromosomal location of *lolCDE* was deleted and the P. aeruginosa *lolCDE* genes were placed in the *ctx* site under the control of the arabinose promoter was employed. Cells were grown in the presence of 0.2% arabinose (+) to an OD_600_ of 0.5. After washing twice with LB broth with no arabinose, the cells were resuspended in medium either with 0.2% (+) or without (−) arabinose. (Right) In the cells that were depleted of LolCDE, antialginate antibody detected much-increased production. (Left) Deletion of the *algU* gene led to no detectable alginate, establishing the specificity of the alginate assay. OprF detected with OprF antibody served as a control for cell extraction for the dot blots.

As an additional check, RT-qPCR analysis of selected genes was performed in P. aeruginosa in which the native *lolCDE* operon of P. aeruginosa was placed under the control of the arabinose promoter. By growing the bacteria in Luria-Bertani (LB) medium supplemented with 0.2% arabinose and then reducing (0.05%) or removing the arabinose, LolCDE levels would diminish over time. The expectation would be that similar changes in expression would be observed in response to compound 2A inhibition of LolCDE function. Levels of RNA were measured by RT-qPCR for several of the identified genes that exhibited significant expression changes upon treatment with compound 2A. As shown in Fig. 4 and Fig. S2, the expression changes of these genes confirmed the results seen in cells treated with compound 2A; namely, the loss of LolCDE function leads to a conserved transcriptional response. Similarly, the levels of alginate measured by dot blot immunoassays were found to increase substantially with the removal of the arabinose inducer and subsequent growth dilution of the native P. aeruginosa LolCDE (Fig. 5).

### Comparison of the effects of compound 2A and antibiotics.

Several of the key genes whose transcript levels were significantly altered by the exposure of PAO1 Δ*mexAB-oprM* Δ*lolCDE_PAO1_ ctx*::*lolCDE_E.coli_* to compound 2A were reconfirmed using RT-qPCR assays. At the same time, we also wished to determine the specificity of the response in relation to several antibiotics with different, known mechanisms of action. MICs were measured in LB medium (used for all experiments) and are presented in Table S4. The P. aeruginosa strain with Δ*mexAB-oprM* and the E. coli *lolCDE* genes in place of its native *lolCDE* was grown for two independent sets of experiment. The cultures were treated for 45 min at an OD at 600 nm (OD_600_) of 0.5 with 3× MIC of compound 2A or with 3× MIC of the indicated antibiotics. The RNAs were extracted and prepared for RT-qPCR. Primers were designed to amplify key genes whose expressions were perturbed by compound 2A. The *proC* gene was employed as a housekeeping gene, whose level of transcription should not vary significantly from that for the control cells, to determine the efficiency and variability of the RNA extractions in the different experiments using cells treated with the LolCDE inhibitor or with various antibiotics.

**FIG 6:**
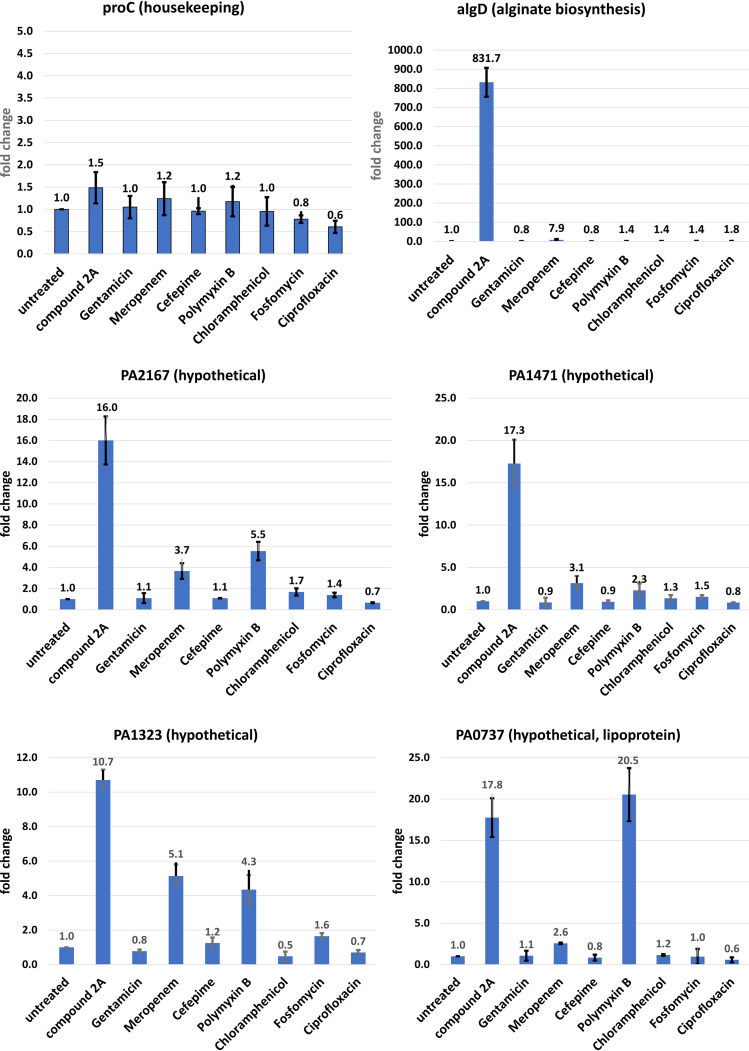

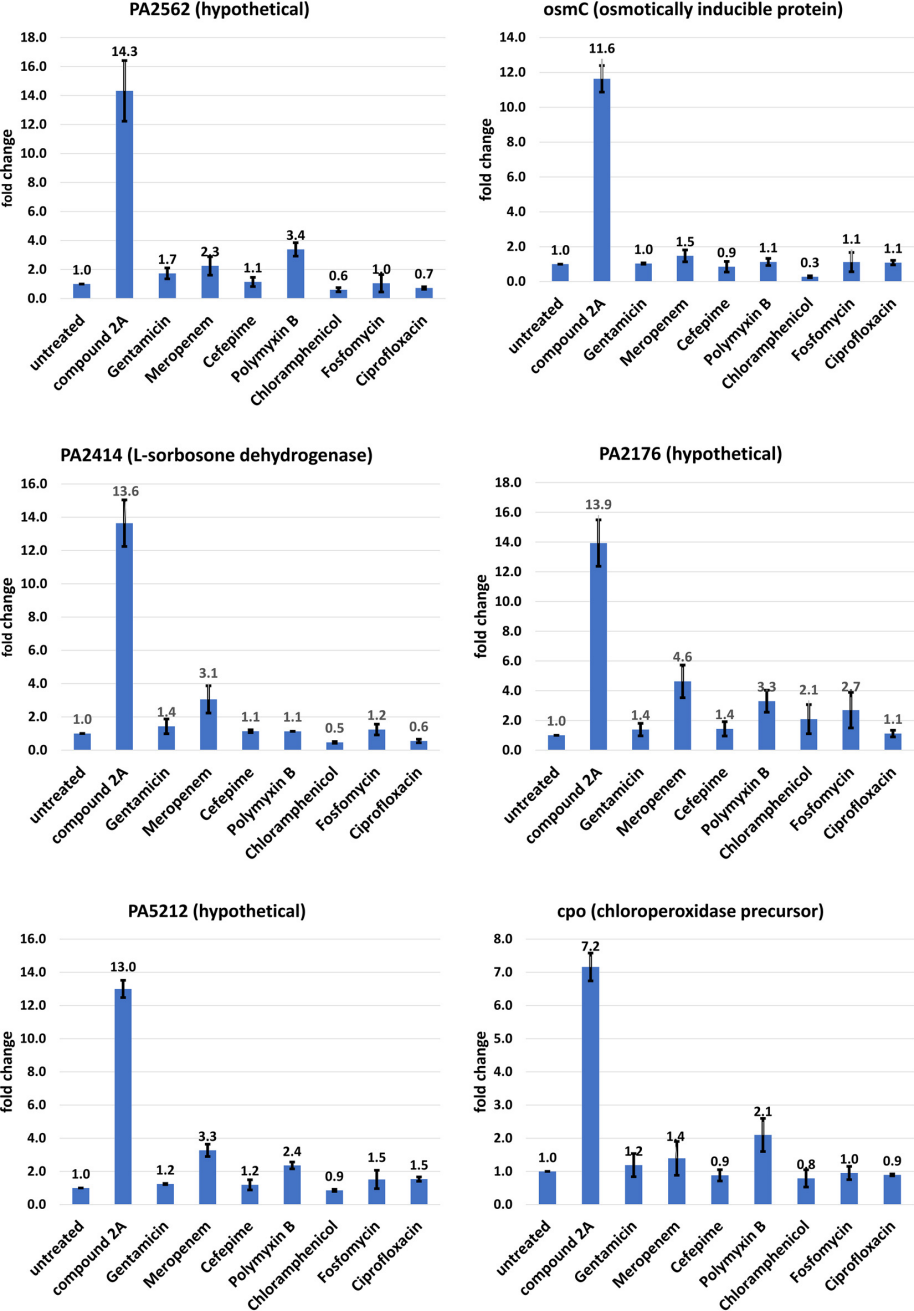

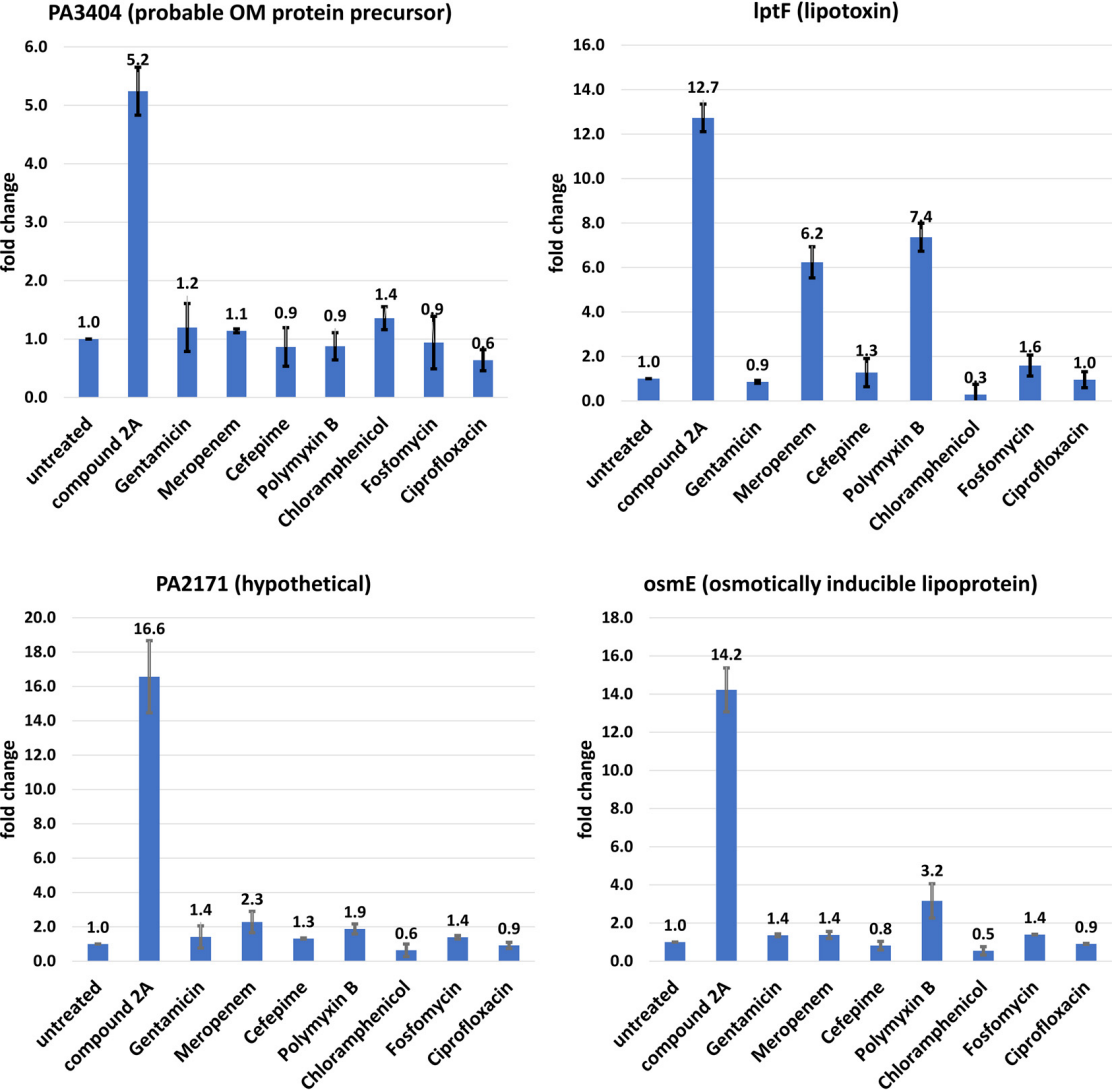
Expression levels of several key genes with inhibitor antibiotics with differing mechanisms of action. Genes that exhibited significantly upregulated changes in the presence of compound 2A were measured by RT-qPCR in the presence of known antibiotics with differing mechanisms of action. Values are the averages from duplicate independent experiments, with standard deviations indicated. The housekeeping gene *proC* was used as a control. Values are relative to those for untreated cells in the first column of each graph. Antibiotics were present at 3× MIC for 45 min in LB broth before RNA extraction. Antibiotic MICs were measured in LB broth, as experiments were all performed in LB broth. MIC results for the compounds are available in Table S4 in the supplemental material.

Figure 6 presents the average results from the two independent RT-qPCR experiments for several key upregulated genes. As far as responses to all the inhibitors, *algD*, *osmC*, PA2171 (hypothetical unknown), and PA3404 (probable OM protein precursor) gave the clearest responses to induction exclusively by compound 2A, and these represent biomarkers of inhibition of lipoprotein transport. In the cases of PA2146, PA2167, PA1471, PA1323, PA2562, PA2414, PA2176, PA5212 (all encoding hypothetical unknown proteins), and the annotated *cpo* and *lptF* genes, while the transcriptional responses were strongly induced by compound 2A, additional responses were observed in cells exposed to meropenem, polymyxin B, or both antibiotics. Open reading frame (ORF) PA0737 (hypothetical unknown) had equivalent responses to compound 2A and polymyxin B.

**FIG 7:**
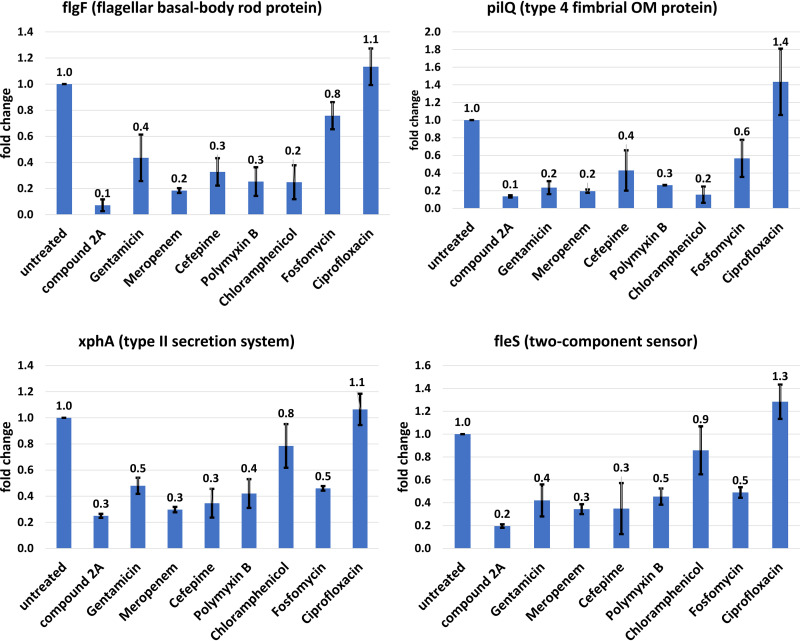
Expression levels of several key genes with inhibitor antibiotics with differing mechanisms of action. Genes that exhibited significant downregulation in the presence of compound 2A were measured by RT-qPCR in the presence of known antibiotics with differing mechanisms of action. Values are the averages from duplicate independent experiments with the indicated standard deviations. Values are relative to those for untreated cells in the first column of each graph. Antibiotics were present at 3× MIC for 45 min in LB broth before RNA extraction.

A less specific response was observed among genes downregulated in the RNA-seq experiments. When a selected group of these transcripts was quantified by RT-qPCR studies, compound 2A was the most potent in reducing their cellular levels; however, several of the other antibiotics had similar effects (Fig. 7). The most notable exception was ciprofloxacin and, to a lesser extent, chloramphenicol. In these cases, the responses to ciprofloxacin matched the untreated control values, while treatment with chloramphenicol showed *xphA* and *fleS* transcripts to be at nearly control levels, and *flgF* and *pilQ* mRNA concentrations were reduced.

## DISCUSSION

Our previous work (23) substituting the E. coli *lolCDE* operon for the native P. aeruginosa version laid the groundwork for the present study of transcriptional responses to LolCDE inhibition by compound 2A. By employing RNA-seq, a number of substantive changes were found to occur in gene expression that could be attributed to the activity of compound 2A on the LolCDE complex; the response very likely reflects cellular sensing of a block in lipoprotein transport and disruption of OM biogenesis. Compared to a similar study in E. coli (17), we find little commonality in the transcriptomes of compound 2-treated cells. Some may arise from the absence of certain genes in each organism; however, where orthologues exist, there was no apparent accumulation or reduction in these mRNAs, indicating that bacteria adapt to specific niches by unique responses.

Most prominent among the genes whose transcript levels were increased in response to compound 2A were those in the alginate pathway. The regulation of alginate synthesis in P. aeruginosa is complex (27). The *mucA* gene forms part of the *algU-mucA-mucB-mucC* operon, which is homologous to the *rpoE-rseA-rseB-rseC* operon in E. coli (28). The *alg* genes are under the control of the stress-related extracytoplasmic function sigma factor (ECF) AlgU; however, it is inactive due to its sequestration by the anti-sigma factor MucA. *alg* operon expression is activated by the degradation of MucA by the AlgW protease or through the acquisition of mutations in MucA, frequently encountered among isolates from individuals with the genetic disease cystic fibrosis (29). Free AlgU directs the expression of *alg* genes by binding to the *algD* promoter, the first gene of the operon (30, 31). Additional regulatory elements control alginate expression, including genes for the two-component regulators AlgR/FimS and AlgB/KinB (32–37). These genes indirectly regulate the expression of *algU* through *algD*. Control may also be exerted at both MucA degradation/AlgU sequestration as well as the induction of the above-described regulators. Since the different alginate regulatory inputs converge on AlgU, and the effect of compound 2A was AlgU dependent (Fig. 2), the activation of any one of the stress-responsive regulators by a defect in lipoprotein transport could lead to the observed increase in alginate production.

The transcription of genes associated with osmotic stress (e.g., *osmC* and *osmE*) increased as well. Both of these genes encode lipoproteins, and their transcription may be increasing due to both osmotic stress and their failure to be transported to their outer membrane locations (38). The lipotoxin F gene *lptF*, also markedly upregulated, is also controlled by the AlgU/T system (39). The chloroperoxidase gene has also been observed to be upregulated by cell wall stress (30). Interestingly, several of the hypothetical genes (PA2167 to PA2173) that showed an upregulated response clustered together, suggesting that an operon response to cell envelope stress may be located in this region. Further work would clarify this possibility.

In addition to the alginate pathway, a number of other transcripts were affected by treatment with compound 2A, including those encoding determinants of flagellum and pilus formation, both of which were substantially downregulated. These effects may be indirectly attributable to compound 2A-induced membrane stress resulting in the downregulation of additional two-component systems, namely, *fleR-fleS* and *pilG-pilH*, resulting in negative effects on the expression of motility and pilus genes, respectively (40, 41). Moreover, the assembly of the type IV pilus utilizes one outer membrane lipoprotein component, PilF (41), while the flagellar basal body components include the lipoprotein FlgH (42, 43). A lack of their transport and, consequently, the formation of defective organelles may be sensed by the regulatory machinery controlling the transcription of genes encoding flagellar or pilus components. This response appears to be specific for flagellum and pilus genes since compound 2A caused an opposite effect on alginate expression; the transport of this polysaccharide also requires a lipoprotein (AlgK), while other transcripts of molecular machineries, such as type II, type III, and type VI secretion systems, each containing the lipoproteins HxcQ (type II), ExsB, PscJ (type III), and TagQ (type IV), were unaffected (44–46).

Although there is strong evidence that compound 2A functions by specifically inhibiting LolCDE, we could not exclude the possibility that the observed changes in transcript levels following treatment with this compound and analyzed by RNA-seq were due to an indirect effect on bacterial physiology and not the consequence of inhibition of lipoprotein transport. After identifying genes with changes in mRNA levels above 10-fold, we confirmed the effect of compound 2A in P. aeruginosa Δ*mexAB-oprM* lacking native *lolCDE* but expressing the E. coli orthologues by RT-qPCR. We then used RT-qPCR to determine the mRNA levels in P. aeruginosa Δ*mexAB-oprM* expressing the native P. aeruginosa *lolCDE* genes under the control of the pBAD promoter and AraC. By reducing the amount of P. aeruginosa LolCDE by limiting the concentration of the inducer (arabinose) in the growth medium, we observed increases in the levels of the same transcripts as those seen in P. aeruginosa *lolCDE_E.coli_* following treatment with compound 2A. In addition, using antibodies to alginate, we have shown that alginate production was upregulated by P. aeruginosa LolCDE depletion. These data reinforce the case that the gene expression changes are an authentic response to interference with LolCDE function.

Several antibiotics with distinct mechanisms of action were tested along with compound 2A for gene expression changes upon exposure. The antibiotics were tested for a limited time (45 min) and at concentrations that minimized extensive cell inhibition and killing. The objective was to identify genes whose expression was changed largely or solely by LolCDE inhibition. These genes could then serve as reporters for the presumptive identification of novel candidate molecules or chemical modifications of compound 2A that would inhibit the native P. aeruginosa LolCDE. Promising candidates in terms of specificity were identified in the responses of *algD*, *osmC*, and ORFs PA2171 and PA3404. Several groups have considered the various steps during the biogenesis of the Gram-negative cell envelope as novel antibiotic targets (18, 47–52). For the screening of lipoprotein transport in P. aeruginosa for a potential therapeutic inhibitor, the development of promoter-reporter constructs with one or more of these genes, employing, for example, β-galactosidase, fluorescent proteins, or luciferase, would serve as a first line of identification in a cell-based screening system for P. aeruginosa LolCDE inhibitors (18, 49, 61). It was interesting to note that antibiotics such as meropenem and polymyxin B, which gave some gene responses overlapping those of the LolCDE inhibitor, are also known to affect aspects of bacterial cell envelope biogenesis. Meropenem has an affinity for *Pseudomonas* penicillin-binding proteins (PBPs) 2, 3, and 4, whereas the other β-lactam antibiotic, cefepime, has a PBP binding profile distinctly different from that of meropenem (53, 54). Disruption of the OM by polymyxins has been well documented (55).

The effects of LolCDE inhibition in both E. coli and P. aeruginosa have now been described (17, 23). In both cases, the two bacteria cease growth and undergo cell lysis in response to LolCDE inhibition. In contrast to the well-defined envelope stress systems of E. coli (19–22), the responses described here for P. aeruginosa are still in the process of being delineated. Clearly, one well-understood aspect is the massive upregulation of alginate biosynthesis. In addition, a number of genes of unknown function were involved in the responses observed. How these changes are connected to the disruption of cell envelope lipoprotein transport will be a topic for future exploration.

## MATERIALS AND METHODS

### Bacterial strains and culture conditions.

P. aeruginosa and E. coli genotypes and plasmids are listed in Table 4. The bacteria were cultured for all experiments in Luria-Bertani (LB) medium at 37°C with shaking at 300 rpm. Antibiotics for genetic selection were used at the following concentrations: tetracycline (Tc) at 30 μg/ml and gentamicin (Gm) at 75 μg/ml for P. aeruginosa and tetracycline at 10 μg/ml, ampicillin (Amp) at 100 μg/ml, and gentamicin at 15 μg/ml for E. coli. Compound 2A (26) (see Fig. S1 in the supplemental material) was obtained in a powder form from AChemtek (Worcester, MA), dissolved in dimethyl sulfoxide to obtain a 5-mg/ml stock, and stored at −20°C.

**TABLE 4:** Strains and plasmids

Strain or plasmid	Genotype or description	Reference or source
Strains		
P. aeruginosa		
PAO1	Wild-type strain	57
PAO1 Δ*mexAB-oprM*	PAO1 with unmarked *mexAB-oprM* deletion	23
PAO1 Δ*lolCDE ctx*::*lolCDE_PAO1_*	*lolCDE* deletion strain with PAO1 *lolCDE* inserted into the CTX phage attachment site (Tc^r^) under the control of the arabinose promoter	23
PAO1 Δ*lolCDE ctx*::*lolCDE_E.coli_*	*lolCDE* deletion strain with E. coli *lolCDE* inserted into the CTX phage under the control of the arabinose promoter	23
PAO1 Δ*mexAB-oprM* Δ*lolCDE ctx*::*lolCDE_PAO1_*	PAO1 Δ*lolCDE*::*lolCDE_PAO1_* with *mexAB-oprM* deletion	23
PAO1 Δ*mexAB-oprM* Δ*lolCDE ctx*::*lolCDE_E.coli_*	PAO1 Δ*lolCDE*::*lolCDE_E.coli_* with *mexAB-oprM* deletion	23
PAO1 Δ*mexAB-oprM* Δ*lolCDE ctx*::*lolCDE_PAO1_* Δ*algU*	*lolCDE* deletion strain with PAO1 *lolCDE* inserted into the CTX phage site; Δ*algU*	This study
PAO1 Δ*mexAB-oprM* Δ*lolCDE ctx*::*lolCDE_E.coli_* Δ*algU*	*lolCDE* deletion strain with E. coli *lolCDE* inserted into the CTX phage site; Δ*algU*	This study
E. coli		
DH5α	F^−^ ϕ80*lacZ*ΔM15 Δ(*lacZYA-argF*)*U169 deoR recA1 endA1 hsdR17*(r_K_^−^ m_K_^+^) *phoA supE44* λ^−^ *thi-1 gyrA96 relA1*	Invitrogen
DH5α/pEXG2-ΔalgU	pEXG2-ΔalgU deletion construct	This study
DH5α/pSW196-lolCDE*_E.coli_*	pSW196-lolCDE*_E. coli_* construct	23
DH5α/pSW196-lolCDE*_p.aeruginosa_*	pSW196-lolCDE*_P. aeruginosa_* construct	23

Plasmids		
pSW196	Site-specific integrative plasmid; pBAD promotor; *attB* (Tc^r^)	58
pEXG2	Allelic-exchange vector (Gm^r^)	59
pRK2013	Helper plasmid with conjugative properties (Km^r^)	60
pSW196-*lolCDE_PAO1_*	pSW196 carrying P. aeruginosa PAO1 *lolCDE*	
pSW196-*lolCDE_E.coli_*	pSW196 carrying E. coli *lolCDE*	
pEXG2Δ*mexAB oprM*	*mexAB-oprM* deletion construct	
pEXG2Δ*algU*	*algU* deletion construct	

### MIC determination.

The MIC values for Δ*mexAB-oprM* P. aeruginosa strains with either P. aeruginosa PAO1 or E. coli *lolCDE* genes at the *ctx* site against compound 2A were determined in microtiter plates (LB broth with 0.2% l-arabinose) at 5 × 10^5^ CFU/ml. The MIC values of gentamicin, meropenem, cefepime, polymyxin B, chloramphenicol, fosfomycin, and ciprofloxacin were determined using Etest strips (bioMérieux Inc.) on LB agar plates with 0.2% l-arabinose and an inoculum of 5 × 10^5^ CFU/ml. MIC values on Mueller-Hinton (MH) agar were within 1 dilution of the LB broth values.

### LolCDE replacement.

The *lolCDE* genes from P. aeruginosa or E. coli were cloned into the EcoRI/SpeI sites of pSW196 under the control of the arabinose-inducible P_BAD_ promoter. Plasmids pSW196-*lolCDE*_PAO1_ and pSW196-*lolCDE_E.coli_* were conjugated into PAO1 using triparental mating with helper plasmid pRK2103. Tetracycline-resistant transconjugants were checked for the genomic insertion of the *lolCDE* genes at the CTX site (56) by PCR with primers flanking the insertion site and subsequent DNA sequencing.

### P. aeruginosa *lolCDE* deletion.

Following the introduction of either the P. aeruginosa or E. coli *lolCDE* genes into the CTX site, for the deletion of the *lolCDE* genes at their original genome locus, ∼500 bp of upstream and downstream regions flanking the native *Pseudomonas lolCDE* genes were cloned into pEXG2 in E. coli. The resulting plasmid, pEXG2 Δ*lolCDE*, was conjugated into the PAO1 strains that have *lolCDE* from P. aeruginosa or E. coli inserted at the CTX site. Transconjugants with a deletion of the native genomic *lolCDE* alleles were selected on medium containing 6% sucrose and 0.5% l-arabinose. Resolved strains were tested for gentamicin sensitivity, and the deletion of the native *lolCDE* locus was confirmed by sequencing a PCR product using primers for the upstream and downstream genes flanking the native *lolCDE* operon. The *lolCDE* deletion strains were dependent on arabinose for viability. Deletions of *mexAB-oprM* and *algU* were done in a similar fashion.

### Transcriptome analysis by RNA-seq.

For transcriptome sequencing (RNA-seq), P. aeruginosa *mexAB-oprM* Δ*lolCDE_PAO1_ ctx*::*lolCDE_E.coli_* was grown overnight in LB broth with 0.2% arabinose with shaking at 37°C. The next morning, a 1:200 dilution was made in 100 ml of LB broth plus 0.2% arabinose, and the bacteria were grown at 37°C with shaking until they reached an optical density at 600 nm (OD_600_) of 0.5. The culture was then split into six portions of 10 ml each that were placed into six flasks, with bacteria in two flasks being used as biological replicate controls, each of two replicate flasks receiving 48 μg/ml of compound 2A, and two replicate flasks receiving 96 μg/ml of the same inhibitor.

After 45 min of exposure to compound 2A, 800 μl of the culture from each flask was placed directly into 800 μl of a prewarmed (65°C) lysis mix-acid phenol solution. Lysis mix consisted of 320 mM sodium acetate, 8% SDS, and 16 mM EDTA (all from Ambion and Thermo Fisher) in nuclease-free water. One hundred microliters of the above-described lysis mix was combined with 700 μl of acid phenol-chloroform (Ambion) in 2-ml tubes. The cells and lysis mix-acid phenol were rapidly mixed on a vortex mixer and kept at 65°C with periodic vortexing for 5 to 10 s every minute for 10 min. RNA isolation, purification, concentrations, and DNase treatment were performed as previously described (17). Ribosome integrity numbers (RINs) were determined with an Agilent Bioanalyzer 2100 instrument and an Agilent RNA 6000 Nano kit. The RINs ranged from 9.8 to 10 for all samples. rRNA depletion was carried out with a RiboMinus kit (Thermo-Fisher). Library preparation for Illumina sequencing was performed as previously described (17). RNA-seq was carried out on an Illumina NextSeq 500 platform. Analysis of the data was performed using CLC Bio Genomics Workbench software, with the reads being mapped to the genome sequence of P. aeruginosa PAO1. Replicates were highly comparable in plots against each other and divergent in plots of controls versus compound treatment (Fig. S3).

### RT-qPCR.

For the determination of RNA levels by reverse transcription-quantitative PCR (RT-qPCR), P. aeruginosa strains were grown with the indicated concentrations of either arabinose (depletion experiments) or antibiotics for either 4 or 6 h. RNA was then prepared from these cultures using the hot acid phenol procedure as described above. Primers were designed by the use of the GenScript real-time PCR primer design tool. cDNA was synthesized with a SuperScript III first-strand synthesis system for reverse transcription-PCR (Invitrogen) and random hexamer primers. RT-qPCR was carried out using PerfeCTa SYBR green FastMix (Quanta Biosciences) in a Mastercycler Realplex2 system from Eppendorf. Changes in transcript levels relative to the levels in the untreated control cultures were calculated. Growth and compound exposures for RNA extractions for the RT-qPCR experiments were performed twice (biological replicates) on different days.

### Alginate antibody dot blots.

A sample of the culture corresponding to 400 μl at an OD_600_ of 1.0 was centrifuged at 12,000 × *g* for 1 min. The supernatant was discarded, the cell pellet was resuspended in 40 μl of 2× Laemmli sample buffer (Bio-Rad) and boiled for 5 min, and 2 μl was pipetted onto a nitrocellulose membrane. The membrane was allowed to air dry (∼30 min), blocked with Tris-buffered saline–Tween 20 (TBST) with 5% skim milk for 1 h, washed three times for 10 min with TBST, and incubated with primary antibody overnight at 4°C. The next day, the filter was washed three times for 10 min each with TBST, incubated with secondary antibody (horseradish peroxidase [HRP] conjugated) for 1 h at room temperature, washed three times for 10 min with TBST, and incubated with ECL chemiluminescent solution for 5 min, and luminescence was detected with X-ray film. The alginate antibody was obtained from Sigma (monoclonal, anti-mouse), and OprF was detected by anti-rabbit antibody, raised in-house.

## Supplementary Material

Supplemental file 1

Supplemental file 2

Supplemental file 3
