# Supplementary material for: Transcriptional Responses of Pseudomonas aeruginosa to Inhibition of Lipoprotein Transport by a Small Molecule Inhibitor
Source: J Bacteriol. 2020 Nov 19;202(24):e00452-20. doi: 10.1128/JB.00452-20 (PMC7685553; doi:10.1128/JB.00452-20)
Supplement: Supplemental file 1 [file JB.00452-20-s0001.pdf]

Supplemental Data
JB-00452-20

Supplemental Data Table 1

Illumina NextSeq 500 Sequencing
NextSeq Mid Output
Single End 150
Total reads: 165,887,558

		Sample reads
Control	A	27,335,904
	B	27,075,496
3 x MIC Compound 2A	A	25,561,763
	B	23,387,502
6 x MIC Compound 2A	A	27,273,839
	B	26,388,822

A and B represent the two duplicate samples in each treatment.

Supplemental Data Table 2

Gene changes for the entire *P. aeruginosa* transcriptome at 3 X MIC of Compound 2A vs. untreated control cells

(Separate Excel file: Supplemental Data Table 2)

Supplemental Data Table 3

Gene expression changes for the entire *P. aeruginosa* transcriptome at 6 X MIC of Compound 2A vs. untreated control cells

(Separate Excel file: Supplemental Data Table 3)

Supplemental Data Table 4

**Compound**	**MIC**	**3 X MIC**
Compound 2A	16 µg/ml	48 µg/ml
Gentamicin	0.18 µg/ml	0.5 µg/ml
Meropenem	0.18 µg/ml	0.5 µg/ml
Cefepime	0.3 µg/ml	1 µg/ml
Polymyxin B	0.75 µg/ml	2 µg/ml
Chloramphenicol	0.18 µg/ml	0.5 µg/ml
Fosfomycin	2.6 µg/ml	8 µg/ml
Ciprofloxacin	0.01 µg/ml	0.03 µg/ml

Antibiotic concentrations for qPCR of key transcripts, determined in LB broth for Compound 2A and by Etest on LB agar for other antibiotics. For the RT-qPCR experiments, 3x MIC was used with 45 minute exposure for the PAO1Δ*mexAB-oprM,* Δ$CDE_{PAO1,}$ *CTX*::$lolCDE_{E.coli}$ strain prior to RNA isolation.

Supplemental Figures
JB00452-20

Fig. S1

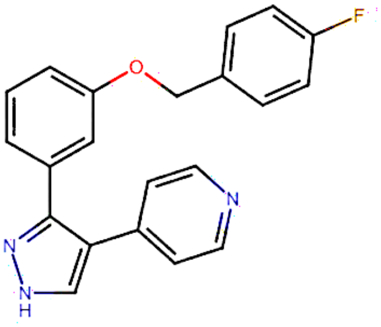

Compound 2

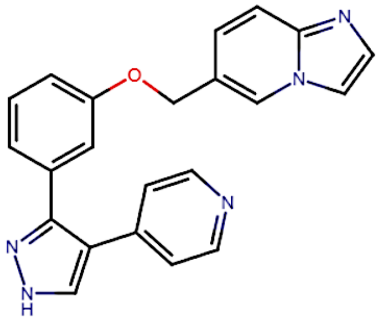

Compound 2A

Supplemental Fig S1. The chemical structures of LolCDE inhibitor Compound 2 and Compound 2A

Fig. S2 **qPCR: PAO1*ΔlolCDE CTX :: lolCDE*$_{PAO1}$ depletion**

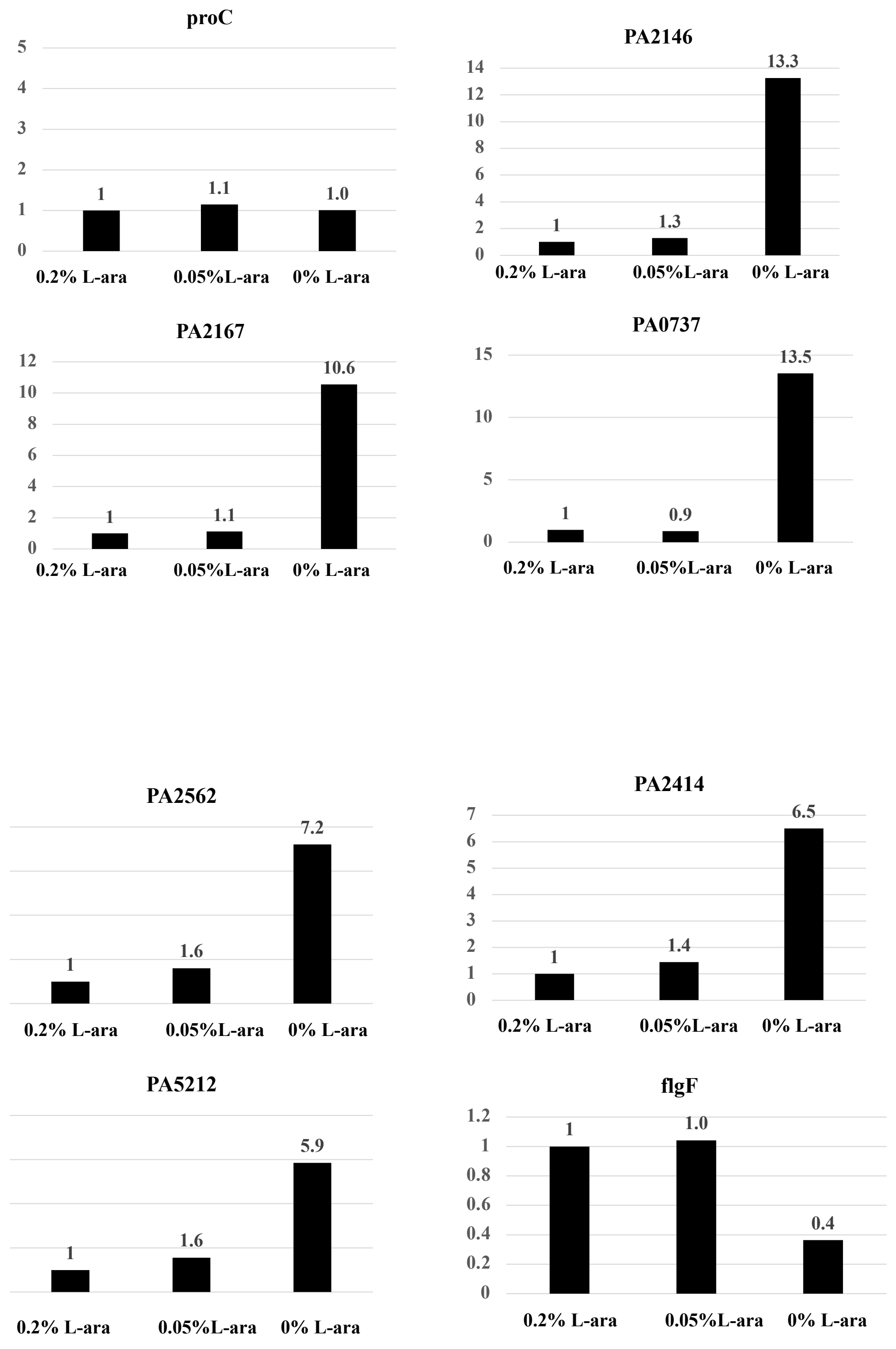

Supplemental Fig. S2**.** Expression of key genes measured by RT-qPCR at 4 hours in 0.2%, 0.05% and no arabinose. The *P. aeruginosa* chromosomal LolCDE genes were deleted after placing the native *P. aeruginosa* LolCDE genes in the *ctx* site under the expression control of

the arabinose promoter system. Cells were grown in 0.2% arabinose then washed and grown in 0.2%, 0.05% or no arabinose for 4 hours before RNA extraction for RT-qPCR. Values are averages of two independent experiments relative to 0.2% arabinose levels, expressed as 1.0 (control value).

Fig. S3

<u>RNA-Seq Scatter plots:</u>

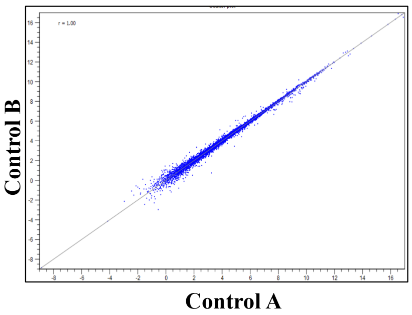

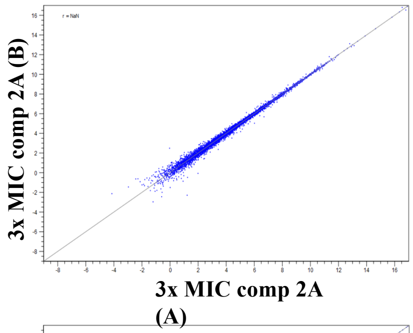

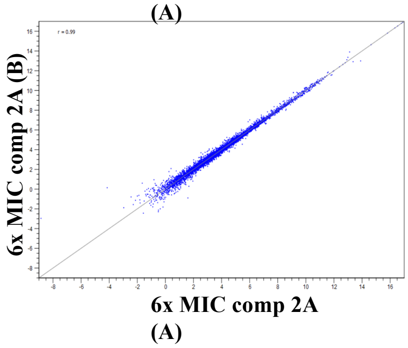

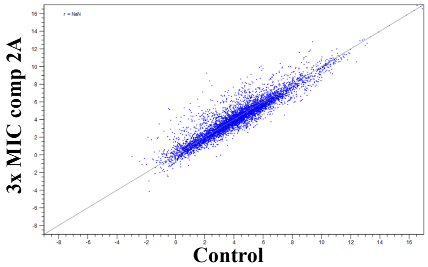

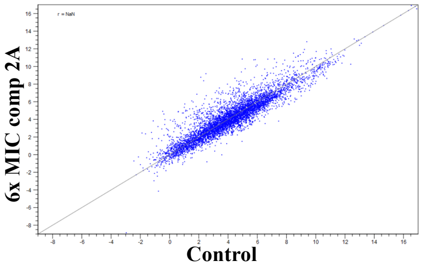

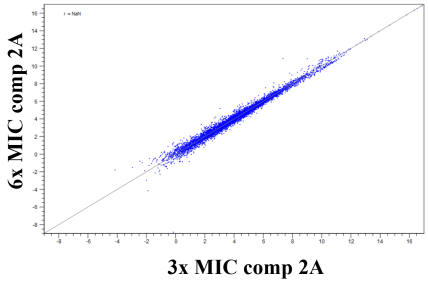

Supplemental Fig. S3. Scatter plots of the RNA-seq results of the duplicate cultures compared to each other, as well as compared to the control, and the 3X and 6X MIC plots. The tight plots of the duplicates indicate the reproducibility of the transcriptional values of the two cultures in the control and two Compound 2A treated cultures. Significant deviations from the line representing transcriptional changes are observed in both the 3 and 6 X MIC plotted against control cultures.
